# RETRACTION: Surgical Therapy vs Conservative Therapy for Patients With Acute Injury of Lateral Ankle Ligament: A Meta‐Analysis and Systematic Review

**DOI:** 10.1111/iwj.70638

**Published:** 2025-04-23

## Abstract

**RETRACTION:** LiuF. Q. and DiaoN. C. , “Surgical Therapy vs Conservative Therapy for Patients With Acute Injury of Lateral Ankle Ligament: A Meta‐Analysis and Systematic Review,” International Wound Journal 16, no. 4 (2019): 925–931, 10.1111/iwj.13120.30924305 PMC7948700

The above article, published online on 28 March 2019, in Wiley Online Library (http://onlinelibrary.wiley.com/), has been retracted by agreement between the journal Editor in Chief, Professor Keith Harding; and John Wiley & Sons Ltd. Following an investigation by the publisher, all parties have concluded that this article was accepted solely on the basis of a compromised peer review process. The editors have therefore decided to retract the article. The authors did not respond to our notice regarding the retraction.

**RETRACTION:** F. Q. Liu and N. C. Diao , “Surgical Therapy vs Conservative Therapy for Patients With Acute Injury of Lateral Ankle Ligament: A Meta‐Analysis and Systematic Review,” International Wound Journal 16, no. 4 (2019): 925–931, 10.1111/iwj.13120.30924305 PMC7948700

The above article, published online on 28 March 2019, in Wiley Online Library (http://onlinelibrary.wiley.com/), has been retracted by agreement between the journal Editor in Chief, Professor Keith Harding; and John Wiley & Sons Ltd. Following an investigation by the publisher, all parties have concluded that this article was accepted solely on the basis of a compromised peer review process. The editors have therefore decided to retract the article. The authors did not respond to our notice regarding the retraction.

